# Epigenetics of Neurodevelopmental Disorders Comes of Age with Roles in Clinical and Educational Applications

**DOI:** 10.3390/ijms19092720

**Published:** 2018-09-12

**Authors:** Takeo Kubota

**Affiliations:** Faculty of Child Studies, Seitoku University, Chiba 271-8555, Japan; takeokubota27@gmail.com

Epigenetics is a gene regulation mechanism that does not depend on genomic DNA sequences, but depends instead on chemical modifications of DNA and histone proteins. The failure of epigenetic mechanisms is known to cause congenital neurodevelopmental disorders (NDs), which include genomic imprinting disorders (e.g., Prader-Willi and Angelman syndrome) [1], X-chromosome inactivation disorders (e.g., ring X Turner syndrome) [2], and epigenetic regulation-associated molecular disorders (e.g., Rett syndrome and Kleefstra syndrome) [3,4]. These indicate that the epigenetic system is essential for normal birth and development.

It has been recently reported that the number of children with NDs has increased in several countries, such as the US, Korea, and Japan, in which environmental factors, rather than genetic factors, are thought to be involved in this increase. Since epigenetic modifications in DNA are more vulnerable than DNA sequences to environmental stressors such as malnutrition, environmental chemicals, and mental stress, especially during the early period of life, one can speculate that current socioenvironmental factors cause acquired NDs via epigenetic alterations in the brain [5,6,7].

The epigenome has a reversible property since it is based on removable residues on genomic DNA. Thus, environmentally induced epigenomic alterations can be potentially restored. In fact, some medicines for psychiatric and epileptic disorders are known to restore an altered epigenome, resulting in the correction of gene expression [8,9,10,11]. Therefore, epigenomic-based preemptive medicine that consists of early detection using epigenomic signatures and early interventions that take advantage of the use of epigenomic reversibility are expected.

Under these circumstances, we are pleased to have this opportunity to compile a special issue entitled “Epigenetics of neurodevelopmental disorders” for the *International Journal of Molecular Sciences*. As a Guest Editor, I would like to thank Prof. Dr. Maurizio Battino, an Editor-in-Chief of this journal, for this great opportunity.

I am very proud of the papers contributed to this section that consists of eight review articles by top-level epigenetic researchers in the world. Through this issue, readers will learn current epigenetic understanding of brain function, congenital NDs, and acquired NDs [12,13,14,15,16,17,18,19]. More precisely, Cariaga-Martínez et al. extensively described epigenetic properties in embryonic stem cells and throughout early development phases [12]. Ma et al. revealed a microRNA involved in the pathogenesis of neonatal hypoxic-ischemic encephalopathy and its therapeutic use with complementary oligonucleotides of this microRNA [13]. Hernandez et al. reported an epigenetic mechanism that regulates thyroid hormone in the brain [14]. Kim et al. and Lepri et al. summarized NDs caused by mutations in histone lysine methylation-related genes and Kabuki syndrome, a representative congenital neurodevelopmental disorder caused by mutations in the *lysine methyltransferase 2D* gene [15,16]. Zapata-Martín Del Campo et al. demonstrated subcellular mechanisms of neuropsychiatric and cardiometabolic disorders induced by environmental exposures in the early stages of life and the underlying neuroendocrine mechanisms [17,18]. Hoffmann et al. discussed the phenotypic contributions of epigenomic responses to early life adversities to major depressive disorders and schizophrenia [19].

I hope this collection of papers will help readers to gain a better understanding of what has been done and what is yet to be done in the field of preemptive medicine of NDs, and how this subject is intimately associated with nursing and education in the early stages of life.
